# Supplementary material for: Intrinsic Disorder in Tetratricopeptide Repeat Proteins
Source: Int J Mol Sci. 2020 May 25;21(10):3709. doi: 10.3390/ijms21103709 (PMC7279152; doi:10.3390/ijms21103709)
Supplement: Supplementary file 1 [file ijms-21-03709-s001.pdf]

# Supplementary Materials

## Intrinsic Disorder in Tetratricopeptide Repeat Proteins

**Nathan W. Van Bibber,[1,†] Cornelia Haerle,[1,†] Roy Khalife,[1,†] Bin Xue,[2] and Vladimir N. Uversky[1,3,4,*]**

[1] Department of Molecular Medicine Morsani College of Medicine, University of South Florida, 12901 Bruce B. Downs Blvd., Tampa, Florida 33612, USA; E-Mails: nvanbibber@mail.usf.edu (N.W.V.B.); chaerle@mail.usf.edu (C.H.); roykhalife@mail.usf.edu (R.H.); vuversky@usf.edu (V.N.U.)

[2] Department of Cell Biology, Microbiology and Molecular Biology, School of Natural Sciences and Mathematics, College of Arts and Sciences, University of South Florida, Tampa, Florida 33620, USA; E-mail: binxue@usf.edu;

[3] USF Health Byrd Alzheimer's Research Institute, Morsani College of Medicine, University of South Florida, 12901 Bruce B. Downs Blvd., Tampa, Florida 33612, USA;

[4] Institute for Biological Instrumentation, Russian Academy of Sciences, Federal Research Center "Pushchino Scientific Center for Biological Research of the Russian Academy of Sciences", 4 Institutskaya St., Pushchino, 142290, Moscow Region, Russia

† These authors contributed equally to this work.

***** Author to whom correspondence should be addressed; E-Mail: vuversky@usf.edu; Tel.: +1-813-974-5816; Fax: +1-813-974-7357.

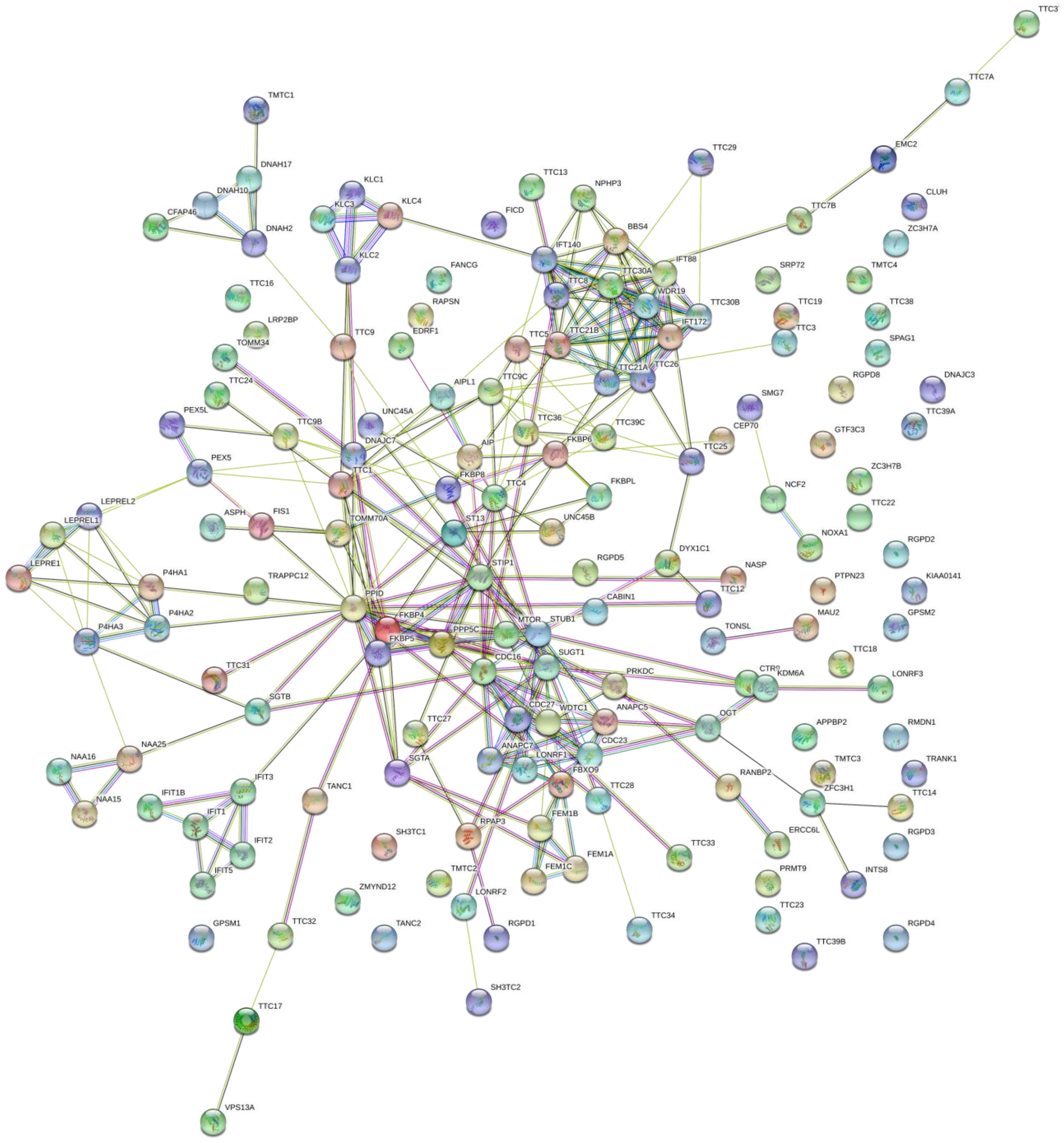


**Supplementary Figure S1**. STRING-generated TPR internal PPI network generated with medium confidence level of 0.4.

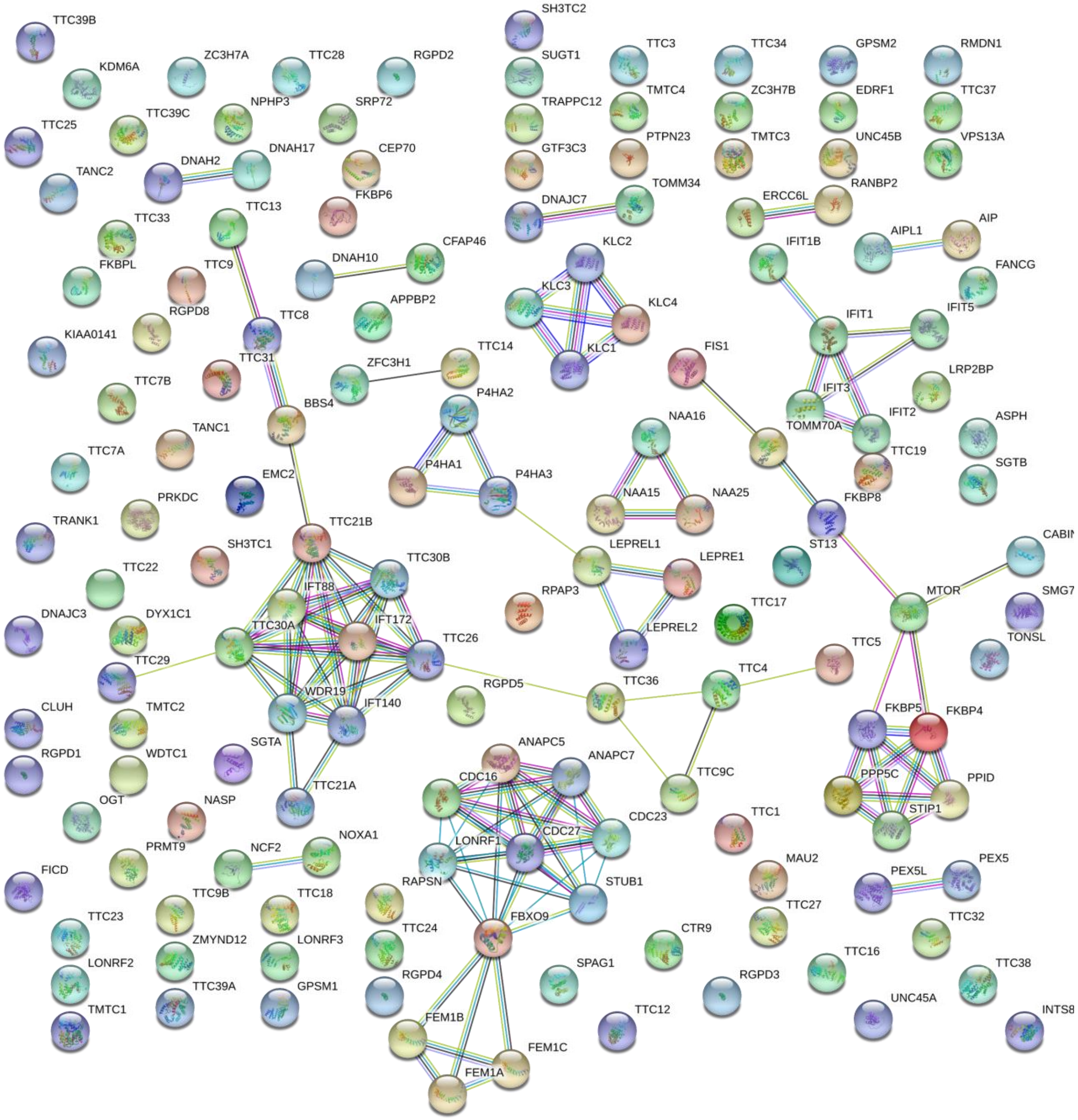


**Supplementary Figure S2**. STRING-generated TPR internal PPI network generated with high confidence of 0.7.

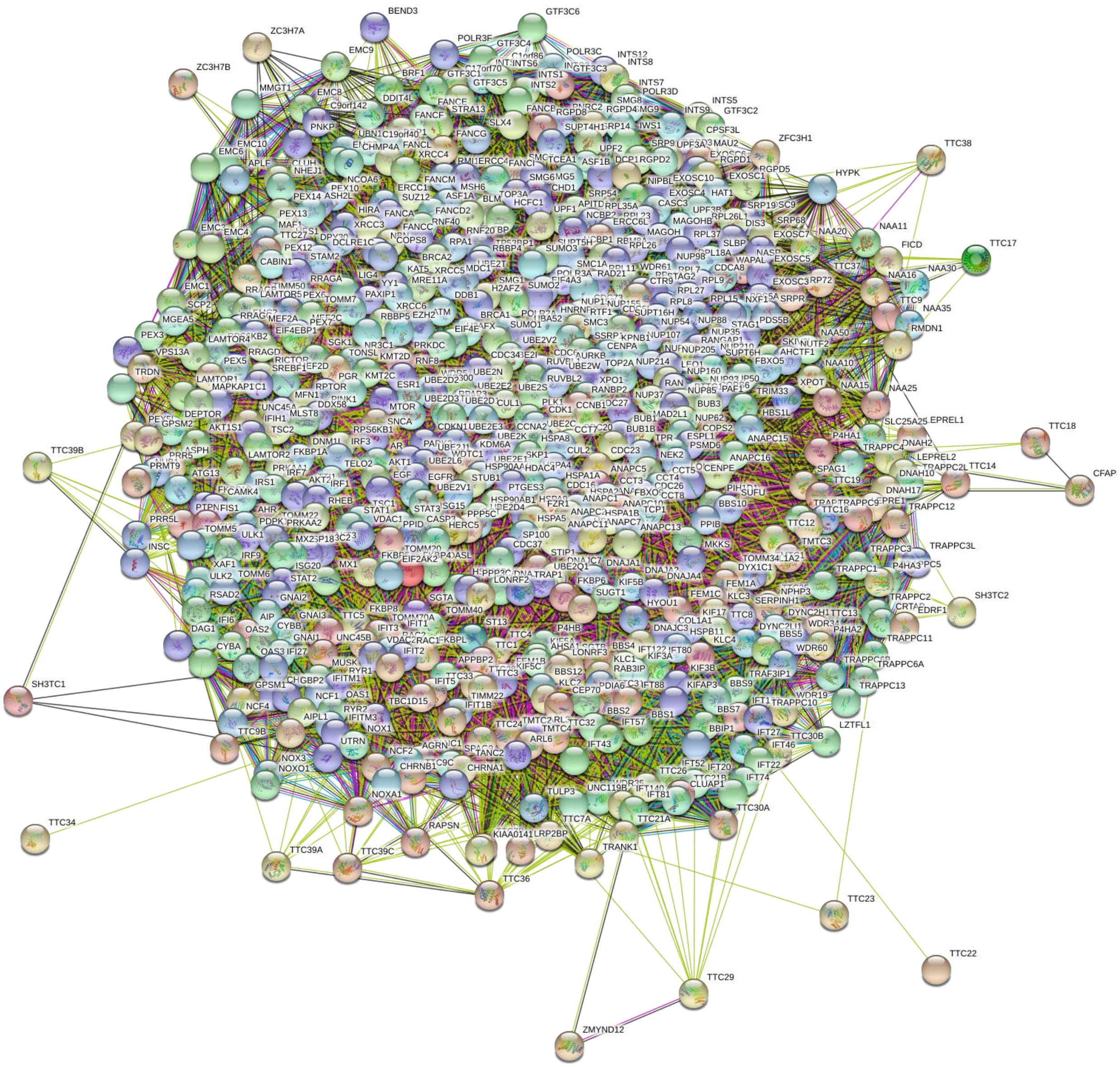


**Supplementary Figure S3**. STRING-generated PPI network of 161 human TPR proteins with included first shell interactors using the low confidence level of 0.15. This dense network of 661 proteins connected by 35,582 interactions with the average node degree of 108, average local clustering coefficient of 0.464, expected number of edges of 23,094, and PPI enrichment p-value of < $10^{-16}$

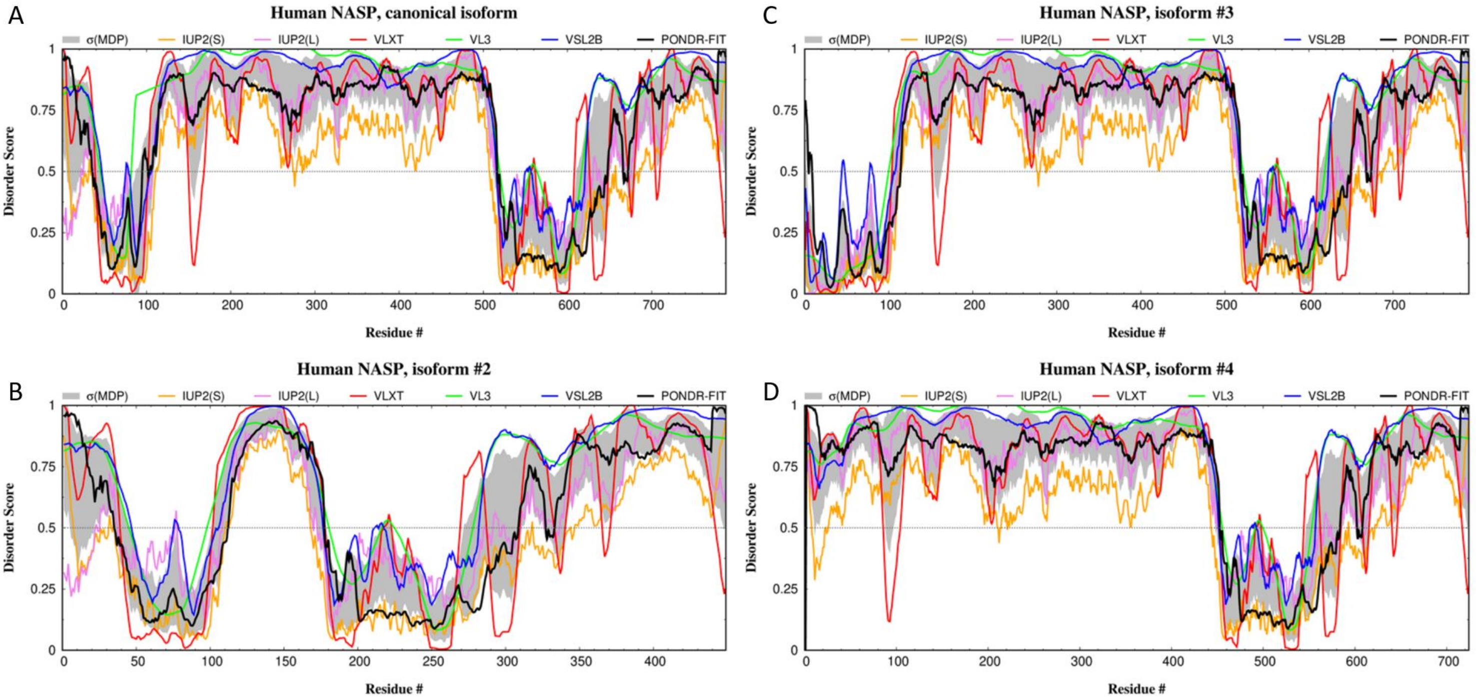


**Supplementary Figure S4**. Intrinsic disorder analysis of alternatively spliced isoforms of human NASP.

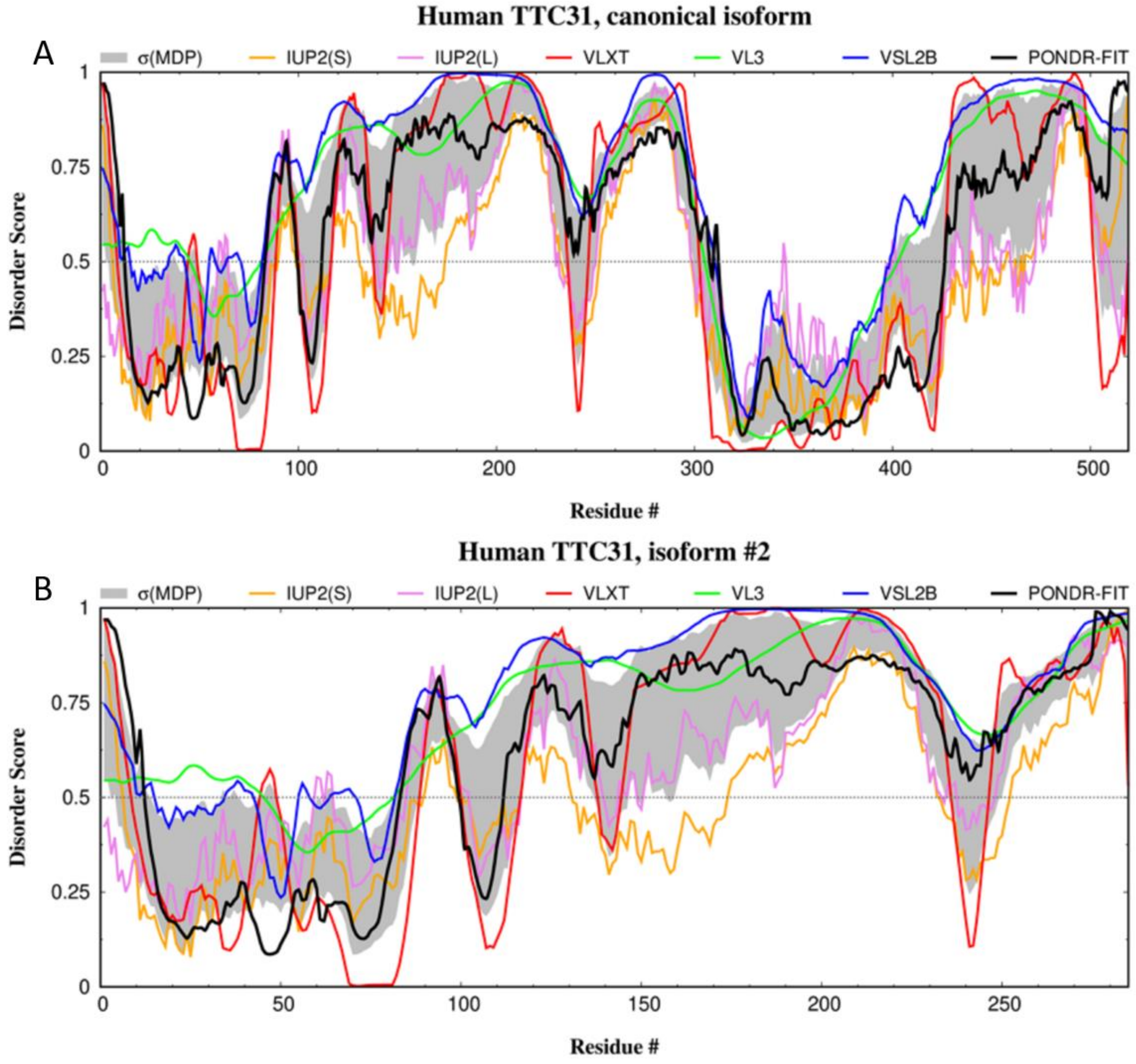


**Supplementary Figure S5**. Intrinsic disorder analysis of alternatively spliced isoforms of human TTC31.

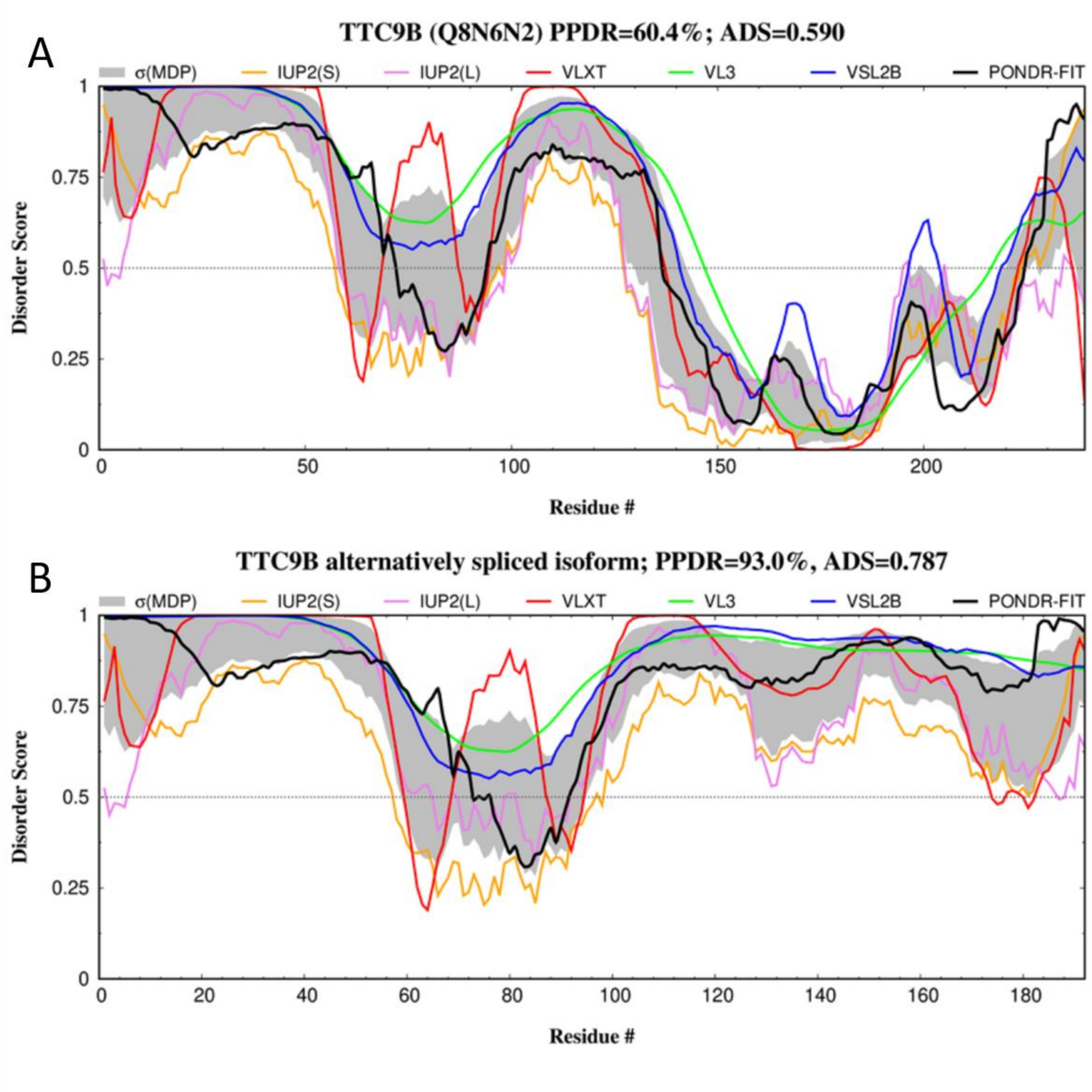


**Supplementary Figure S6**. Intrinsic disorder analysis of alternatively spliced isoforms of human TTC9B.

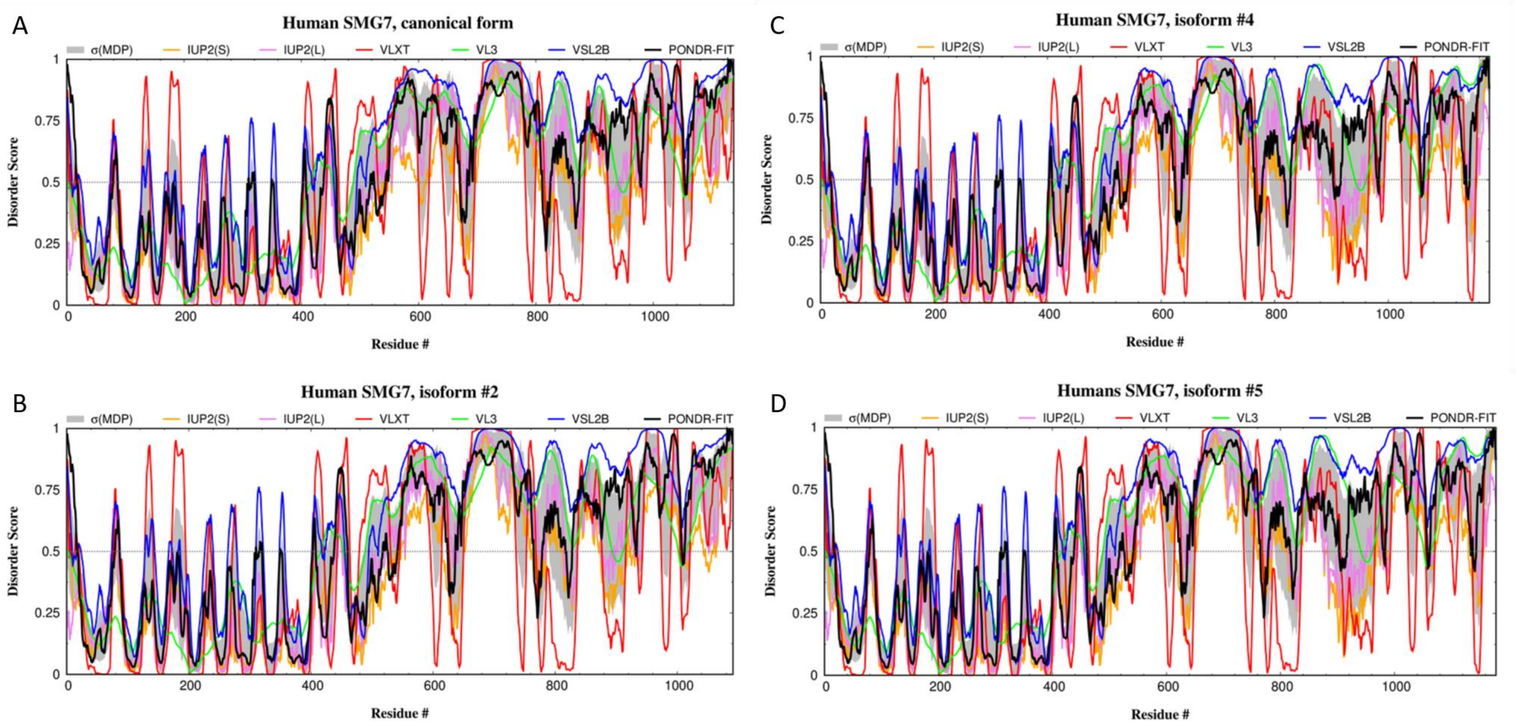


**Supplementary Figure S7**. Intrinsic disorder analysis of alternatively spliced isoforms of human SNG7.

**Supplementary Materials.** 166 human TPR proteins analyzed in this study, their intrinsic disorder propensity, presence of experimentally validated structural information and relation to various human diseases.

| UniProt ID | Protein name | Entry name | Length | PONDR-FIT (% Disordered) | PONDR-FIT (avg.) | VLXT.Overall percent disordered | VLXT.Average Prediction Score | VSL2.Overall percent disordered | VSL2.Average Prediction Score | Cross-reference (PDB) | Structure (PDB) available? | Involvement in disease | Diseases? | Mutagenesis | Mutations recorded? |
|---|---|---|---|---|---|---|---|---|---|---|---|---|---|---|---|
| O00170 | AIP | AIP_HUMAN | 330 | 0.1273 | 0.2426 | 0.2303 | 0.2842 | 0.2545 | 0.3751 | 2LKN;4AIF;4A | TRUE | DISEASE: Pituit | TRUE | | FALSE |
| Q9NZN9 | AIPL1 | AIPL1_HUMAN | 384 | 0.2578 | 0.3217 | 0.2839 | 0.3570 | 0.3307 | 0.4524 | 5U9A;5U9I;5U | TRUE | DISEASE: Leber | TRUE | MUTAGEN 53 53 | TRUE |
| Q6P2P2 | ANM9 | ANM9_HUMAN | 845 | 0.0734 | 0.2053 | 0.1858 | 0.2404 | 0.1680 | 0.3102 | 6PDM; | TRUE | | FALSE | MUTAGEN 182 1 | TRUE |
| Q92624 | APBP2 | APBP2_HUMAN | 585 | 0.0342 | 0.1566 | 0.0786 | 0.1379 | 0.1231 | 0.2934 | | FALSE | | FALSE | | FALSE |
| Q9UJX4 | APC5 | APC5_HUMAN | 755 | 0.1325 | 0.2152 | 0.1642 | 0.2186 | 0.2026 | 0.3523 | 4UI9;5A31;5G | TRUE | | FALSE | | FALSE |
| Q9UJX3 | APC7 | APC7_HUMAN | 599 | 0.1619 | 0.2684 | 0.2137 | 0.3111 | 0.2421 | 0.3920 | 3FFL;4UI9;5A | TRUE | | FALSE | MUTAGEN 56 56 | TRUE |
| Q12797 | ASPH | ASPH_HUMAN | 758 | 0.4393 | 0.4592 | 0.5145 | 0.4870 | 0.5343 | 0.5742 | 5APA;5JQY;5J | TRUE | DISEASE: Facia | TRUE | MUTAGEN 91 93 | TRUE |
| Q96RK4 | BBS4 | BBS4_HUMAN | 519 | 0.2062 | 0.2899 | 0.2389 | 0.2451 | 0.3256 | 0.4051 | | FALSE | DISEASE: Bard | TRUE | | FALSE |
| Q9Y6J0 | CABIN | CABIN_HUMAN | 2220 | 0.3851 | 0.4198 | 0.4851 | 0.4617 | 0.5266 | 0.5592 | 1N6J; | TRUE | | FALSE | MUTAGEN 2172 | TRUE |
| Q13042 | CDC16 | CDC16_HUMAN | 620 | 0.1113 | 0.2198 | 0.1823 | 0.2161 | 0.2403 | 0.3634 | 3HYM;4UI9;5 | TRUE | | FALSE | | FALSE |
| Q9UJX2 | CDC23 | CDC23_HUMAN | 597 | 0.0536 | 0.1941 | 0.1608 | 0.2064 | 0.2278 | 0.3230 | 4UI9;5A31;5G | TRUE | | FALSE | MUTAGEN 339 3 | TRUE |
| P30260 | CDC27 | CDC27_HUMAN | 824 | 0.3726 | 0.3765 | 0.3422 | 0.3433 | 0.4551 | 0.4969 | 3T1N;4RG6;4 | TRUE | | FALSE | MUTAGEN 821 8 | TRUE |
| Q8NHQ1 | CEP70 | CEP70_HUMAN | 597 | 0.4188 | 0.4050 | 0.3920 | 0.3826 | 0.5008 | 0.5433 | | FALSE | | FALSE | | FALSE |
| Q8IYW2 | CFA46 | CFA46_HUMAN | 2715 | 0.1882 | 0.2891 | 0.3506 | 0.3689 | 0.3650 | 0.4343 | | FALSE | | FALSE | | FALSE |
| Q5T0N1 | CFA70 | CFA70_HUMAN | 1121 | 0.1454 | 0.2575 | 0.2596 | 0.2979 | 0.3426 | 0.4073 | | FALSE | | FALSE | | FALSE |
| Q9UNE7 | CHIP | CHIP_HUMAN | 303 | 0.3432 | 0.3806 | 0.4686 | 0.5032 | 0.4191 | 0.5161 | 4KBQ; | TRUE | DISEASE: Spinc | TRUE | MUTAGEN 30 30 | TRUE |
| O75153 | CLU | CLU_HUMAN | 1309 | 0.1551 | 0.2635 | 0.3231 | 0.3401 | 0.2743 | 0.4081 | | FALSE | | FALSE | | FALSE |
| Q6PD62 | CTR9 | CTR9_HUMAN | 1173 | 0.3316 | 0.3810 | 0.3649 | 0.3808 | 0.3777 | 0.5080 | 5ZYQ;6GMH; | TRUE | | FALSE | | FALSE |
| Q8WXU2 | DAAF4 | DAAF4_HUMAN | 420 | 0.3214 | 0.3709 | 0.4167 | 0.3967 | 0.5357 | 0.5354 | | FALSE | DISEASE: Dysle | TRUE | | FALSE |
| Q14154 | DELE1 | DELE1_HUMAN | 515 | 0.3903 | 0.4311 | 0.3650 | 0.3800 | 0.5981 | 0.5916 | | FALSE | | FALSE | | FALSE |
| Q13217 | DNJC3 | DNJC3_HUMAN | 504 | 0.2857 | 0.3614 | 0.3373 | 0.3471 | 0.4881 | 0.5121 | 2Y4T;2Y4U; | TRUE | DISEASE: Ataxi | TRUE | | FALSE |
| Q99615 | DNJC7 | DNJC7_HUMAN | 494 | 0.2085 | 0.3071 | 0.2429 | 0.2698 | 0.4757 | 0.5000 | | FALSE | | FALSE | MUTAGEN 101 1 | TRUE |
| Q8IVF4 | DYH10 | DYH10_HUMAN | 4471 | 0.0541 | 0.1779 | 0.1930 | 0.2541 | 0.1682 | 0.3148 | | FALSE | | FALSE | | FALSE |
| Q9UFH2 | DYH17 | DYH17_HUMAN | 4462 | 0.0193 | 0.1592 | 0.1690 | 0.2297 | 0.1544 | 0.3034 | | FALSE | | FALSE | | FALSE |
| Q9P225 | DYH2 | DYH2_HUMAN | 4427 | 0.0490 | 0.1807 | 0.2254 | 0.2695 | 0.1653 | 0.3150 | | FALSE | | FALSE | | FALSE |
| Q3B7T1 | EDRF1 | EDRF1_HUMAN | 1238 | 0.1963 | 0.3047 | 0.2528 | 0.3115 | 0.4063 | 0.4581 | | FALSE | | FALSE | | FALSE |
| Q15006 | EMC2 | EMC2_HUMAN | 297 | 0.1852 | 0.2884 | 0.2896 | 0.3266 | 0.2929 | 0.4055 | | FALSE | | FALSE | | FALSE |
| Q2NKX8 | ERC6L | ERC6L_HUMAN | 1250 | 0.3280 | 0.3785 | 0.3472 | 0.3833 | 0.5128 | 0.5311 | 5JNO; | TRUE | | FALSE | MUTAGEN 11 11 | TRUE |
| P50502 | F10A1 | F10A1_HUMAN | 369 | 0.6125 | 0.5783 | 0.5257 | 0.5711 | 0.7344 | 0.6983 | 1UZS; | TRUE | | FALSE | | FALSE |
| Q8NFI4 | F10A5 | F10A5_HUMAN | 369 | 0.5962 | 0.5527 | 0.4959 | 0.5417 | 0.6992 | 0.6742 | | FALSE | | FALSE | | FALSE |
| O15287 | FANCG | FANCG_HUMAN | 622 | 0.0756 | 0.2475 | 0.3023 | 0.3418 | 0.3505 | 0.4095 | | FALSE | DISEASE: Fanc | TRUE | MUTAGEN 7 7 S | TRUE |
| Q9UK97 | FBX9 | FBX9_HUMAN | 447 | 0.2416 | 0.3089 | 0.2260 | 0.2866 | 0.3445 | 0.4024 | | FALSE | | FALSE | | FALSE |
| Q9BSK4 | FEM1A | FEM1A_HUMAN | 669 | 0.1390 | 0.2714 | 0.3169 | 0.3612 | 0.2571 | 0.3932 | | FALSE | | FALSE | | FALSE |
| Q9UK73 | FEM1B | FEM1B_HUMAN | 627 | 0.0335 | 0.1724 | 0.1116 | 0.1915 | 0.1388 | 0.2846 | | FALSE | | FALSE | MUTAGEN 342 3 | TRUE |
| Q96JP0 | FEM1C | FEM1C_HUMAN | 617 | 0.0567 | 0.1987 | 0.1410 | 0.2170 | 0.1750 | 0.3250 | | FALSE | | FALSE | | FALSE |
| Q9BVA6 | FICD | FICD_HUMAN | 458 | 0.1070 | 0.2219 | 0.2817 | 0.3413 | 0.2489 | 0.3474 | 4U04;4U07;4 | TRUE | | FALSE | MUTAGEN 76 77 | TRUE |
| Q9Y3D6 | FIS1 | FIS1_HUMAN | 152 | 0.1908 | 0.3315 | 0.2895 | 0.2993 | 0.3224 | 0.4121 | 1NZN;1PC2; | TRUE | | FALSE | MUTAGEN 14 14 | TRUE |
| Q02790 | FKBP4 | FKBP4_HUMAN | 459 | 0.1808 | 0.3324 | 0.4009 | 0.3885 | 0.4183 | 0.5026 | 1N1A;1P5Q;1 | TRUE | | FALSE | MUTAGEN 67 68 | TRUE |
| Q13451 | FKBP5 | FKBP5_HUMAN | 457 | 0.2626 | 0.3295 | 0.3195 | 0.3433 | 0.4661 | 0.4993 | 1KT0;3O5D;3 | TRUE | | FALSE | | FALSE |
| O75344 | FKBP6 | FKBP6_HUMAN | 327 | 0.0979 | 0.2107 | 0.1835 | 0.2821 | 0.1407 | 0.3246 | 3B7X; | TRUE | DISEASE: Note | TRUE | | FALSE |
| Q14318 | FKBP8 | FKBP8_HUMAN | 412 | 0.3058 | 0.3832 | 0.4903 | 0.5052 | 0.3981 | 0.4990 | 2AWG;2D9F;2 | TRUE | | FALSE | MUTAGEN 149 1 | TRUE |
| Q9UIM3 | FKBPL | FKBPL_HUMAN | 349 | 0.3868 | 0.4487 | 0.5501 | 0.5045 | 0.6791 | 0.6219 | | FALSE | | FALSE | MUTAGEN 287 2 | TRUE |
| Q86YR5 | GPSM1 | GPSM1_HUMAN | 675 | 0.4119 | 0.4193 | 0.3837 | 0.4076 | 0.5156 | 0.5539 | | FALSE | | FALSE | | FALSE |

| | | | | | | | | | | | | | | | |
|---|---|---|---|---|---|---|---|---|---|---|---|---|---|---|---|
| P81274 | GPSM2 | GPSM2_HUMAN | 684 | 0.2939 | 0.3574 | 0.2982 | 0.3391 | 0.5000 | 0.5001 | 3SF4;4WND;4 | TRUE | DISEASE: Chud | TRUE | MUTAGEN 228 2 | TRUE |
| Q96RY7 | IF140 | IF140_HUMAN | 1462 | 0.0684 | 0.2160 | 0.2298 | 0.2785 | 0.2346 | 0.3654 | | FALSE | DISEASE: Short | TRUE | | FALSE |
| Q9UG01 | IF172 | IF172_HUMAN | 1749 | 0.0212 | 0.1668 | 0.1687 | 0.2450 | 0.1366 | 0.3092 | | FALSE | DISEASE: Short | TRUE | | FALSE |
| P09914 | IFIT1 | IFIT1_HUMAN | 478 | 0.1611 | 0.2633 | 0.2343 | 0.2845 | 0.2113 | 0.3684 | 4HOU;5UDI;5 | TRUE | | FALSE | MUTAGEN 34 34 | TRUE |
| P09913 | IFIT2 | IFIT2_HUMAN | 472 | 0.2267 | 0.3019 | 0.3199 | 0.3260 | 0.4089 | 0.4622 | 4G1T; | TRUE | | FALSE | MUTAGEN 184 1 | TRUE |
| O14879 | IFIT3 | IFIT3_HUMAN | 490 | 0.1347 | 0.2862 | 0.2694 | 0.3104 | 0.3980 | 0.4608 | 6C6K; | TRUE | | FALSE | | FALSE |
| Q13325 | IFIT5 | IFIT5_HUMAN | 482 | 0.0436 | 0.2099 | 0.1390 | 0.2163 | 0.2220 | 0.3608 | 3ZGQ;4HOQ;4 | TRUE | | FALSE | MUTAGEN 33 33 | TRUE |
| Q5T764 | IFT1B | IFT1B_HUMAN | 474 | 0.0527 | 0.2036 | 0.0907 | 0.2058 | 0.2384 | 0.3466 | | FALSE | | FALSE | | FALSE |
| A0AVF1 | IFT56 | IFT56_HUMAN | 554 | 0.1029 | 0.2201 | 0.1733 | 0.2216 | 0.1913 | 0.3113 | | FALSE | | FALSE | | FALSE |
| Q13099 | IFT88 | IFT88_HUMAN | 833 | 0.2449 | 0.3206 | 0.3181 | 0.3559 | 0.3878 | 0.4520 | | FALSE | | FALSE | | FALSE |
| Q75QN2 | INT8 | INT8_HUMAN | 995 | 0.0864 | 0.2077 | 0.2161 | 0.2709 | 0.2221 | 0.3223 | | FALSE | | FALSE | | FALSE |
| O15550 | KDM6A | KDM6A_HUMAN | 1401 | 0.3961 | 0.4124 | 0.3526 | 0.3714 | 0.5139 | 0.5196 | 3AVR;3AVS;6I | TRUE | DISEASE: Kabu | TRUE | MUTAGEN 1146 | TRUE |
| Q07866 | KLC1 | KLC1_HUMAN | 573 | 0.4258 | 0.4194 | 0.4206 | 0.4212 | 0.6038 | 0.5845 | 3NF1;5OJ8; | TRUE | | FALSE | MUTAGEN 521 5 | TRUE |
| Q9H0B6 | KLC2 | KLC2_HUMAN | 622 | 0.4148 | 0.4686 | 0.5997 | 0.5521 | 0.5804 | 0.6000 | 3CEQ;3EDT; | TRUE | DISEASE: Spast | TRUE | | FALSE |
| Q6P597 | KLC3 | KLC3_HUMAN | 504 | 0.4425 | 0.4743 | 0.6171 | 0.5539 | 0.6567 | 0.6138 | | FALSE | | FALSE | | FALSE |
| Q9NSK0 | KLC4 | KLC4_HUMAN | 619 | 0.4701 | 0.4755 | 0.6252 | 0.5614 | 0.6220 | 0.6082 | | FALSE | | FALSE | | FALSE |
| Q17RB8 | LONF1 | LONF1_HUMAN | 773 | 0.1966 | 0.2944 | 0.2678 | 0.3098 | 0.3816 | 0.4472 | | FALSE | | FALSE | | FALSE |
| Q1L5Z9 | LONF2 | LONF2_HUMAN | 754 | 0.3382 | 0.3855 | 0.3501 | 0.3930 | 0.5265 | 0.5533 | | FALSE | | FALSE | | FALSE |
| Q496Y0 | LONF3 | LONF3_HUMAN | 759 | 0.3478 | 0.3930 | 0.3860 | 0.4118 | 0.4980 | 0.5335 | | FALSE | | FALSE | | FALSE |
| Q9P2M1 | LR2BP | LR2BP_HUMAN | 347 | 0.0749 | 0.1860 | 0.1153 | 0.1674 | 0.1470 | 0.3267 | | FALSE | | FALSE | | FALSE |
| P42345 | MTOR | MTOR_HUMAN | 2549 | 0.0891 | 0.2116 | 0.2523 | 0.2981 | 0.1962 | 0.3296 | 1AUE;1FAP;1I | TRUE | DISEASE: Smith | TRUE | MUTAGEN 2159 | TRUE |
| Q9BXJ9 | NAA15 | NAA15_HUMAN | 866 | 0.1201 | 0.2110 | 0.1998 | 0.2518 | 0.2009 | 0.3723 | 6C95;6C9M; | TRUE | DISEASE: Ment | TRUE | | FALSE |
| Q6N069 | NAA16 | NAA16_HUMAN | 864 | 0.1088 | 0.2004 | 0.1806 | 0.2366 | 0.1898 | 0.3721 | | FALSE | | FALSE | | FALSE |
| Q14CX7 | NAA25 | NAA25_HUMAN | 972 | 0.0823 | 0.2260 | 0.2541 | 0.2781 | 0.3200 | 0.3818 | | FALSE | | FALSE | | FALSE |
| P49321 | NASP | NASP_HUMAN | 788 | 0.7487 | 0.6750 | 0.7322 | 0.6654 | 0.8020 | 0.7929 | | FALSE | | FALSE | | FALSE |
| P19878 | NCF2 | NCF2_HUMAN | 526 | 0.2357 | 0.3235 | 0.3270 | 0.3368 | 0.4468 | 0.4567 | 1E96;1HH8;1I | TRUE | DISEASE: Gran | TRUE | | FALSE |
| Q86UR1 | NOXA1 | NOXA1_HUMAN | 476 | 0.2689 | 0.3410 | 0.3782 | 0.3808 | 0.4160 | 0.4636 | | FALSE | | FALSE | MUTAGEN 34 34 | TRUE |
| Q7Z494 | NPHP3 | NPHP3_HUMAN | 1330 | 0.1368 | 0.2527 | 0.2812 | 0.3148 | 0.3789 | 0.4151 | 5L7K; | TRUE | DISEASE: Neph | TRUE | | FALSE |
| O15294 | OGT1 | OGT1_HUMAN | 1046 | 0.0459 | 0.1726 | 0.1606 | 0.2190 | 0.1205 | 0.2957 | 1W3B;3PE3;3 | TRUE | DISEASE: Note | TRUE | MUTAGEN 208 2 | TRUE |
| Q32P28 | P3H1 | P3H1_HUMAN | 736 | 0.1685 | 0.2784 | 0.3220 | 0.3528 | 0.3641 | 0.4177 | | FALSE | DISEASE: Oste | TRUE | | FALSE |
| Q8IVL5 | P3H2 | P3H2_HUMAN | 708 | 0.1695 | 0.2603 | 0.3051 | 0.3391 | 0.3404 | 0.4303 | | FALSE | DISEASE: Myop | TRUE | | FALSE |
| Q8IVL6 | P3H3 | P3H3_HUMAN | 736 | 0.2473 | 0.3618 | 0.4049 | 0.4418 | 0.4891 | 0.5419 | | FALSE | | FALSE | | FALSE |
| P13674 | P4HA1 | P4HA1_HUMAN | 534 | 0.0805 | 0.2106 | 0.2154 | 0.2681 | 0.2041 | 0.3630 | 1TJC;2V5F;2Y | TRUE | | FALSE | MUTAGEN 210 2 | TRUE |
| O15460 | P4HA2 | P4HA2_HUMAN | 535 | 0.0523 | 0.2058 | 0.1963 | 0.2764 | 0.2131 | 0.3514 | 6EVL;6EVM;6 | TRUE | DISEASE: Myop | TRUE | | FALSE |
| Q7Z4N8 | P4HA3 | P4HA3_HUMAN | 544 | 0.0551 | 0.1999 | 0.1728 | 0.2507 | 0.1967 | 0.3317 | | FALSE | | FALSE | | FALSE |
| P50542 | PEX5 | PEX5_HUMAN | 639 | 0.1408 | 0.2863 | 0.3302 | 0.3722 | 0.4163 | 0.4546 | 1FCH;2C0L;2C | TRUE | DISEASE: Pero | TRUE | MUTAGEN 118 1 | TRUE |
| Q8IYB4 | PEX5R | PEX5R_HUMAN | 626 | 0.4329 | 0.4433 | 0.5128 | 0.4836 | 0.5815 | 0.5577 | | FALSE | | FALSE | | FALSE |
| Q08752 | PPID | PPID_HUMAN | 370 | 0.1486 | 0.2768 | 0.1486 | 0.2396 | 0.3541 | 0.4386 | | FALSE | | FALSE | MUTAGEN 227 2 | TRUE |
| P53041 | PPP5 | PPP5_HUMAN | 499 | 0.0882 | 0.2153 | 0.1824 | 0.2470 | 0.2725 | 0.3836 | 1A17;1S95;1V | TRUE | | FALSE | MUTAGEN 32 32 | TRUE |
| P78527 | PRKDC | PRKDC_HUMAN | 4128 | 0.0964 | 0.2064 | 0.1860 | 0.2422 | 0.2202 | 0.3470 | 5LUQ;5W1R;5 | TRUE | DISEASE: Immu | TRUE | MUTAGEN 1510 | TRUE |
| Q9H3S7 | PTN23 | PTN23_HUMAN | 1636 | 0.4034 | 0.4429 | 0.4927 | 0.5187 | 0.5770 | 0.6027 | 3RAU;5CRU;5 | TRUE | | FALSE | MUTAGEN 202 2 | TRUE |
| Q13702 | RAPSN | RAPSN_HUMAN | 412 | 0.0874 | 0.1957 | 0.1505 | 0.2457 | 0.1262 | 0.3408 | | FALSE | DISEASE: Myas | TRUE | | FALSE |
| P49792 | RBP2 | RBP2_HUMAN | 3224 | 0.3294 | 0.3779 | 0.2847 | 0.3204 | 0.6104 | 0.5694 | 1RRP;1XKE;1Z | TRUE | DISEASE: Ence | TRUE | MUTAGEN 2632 | TRUE |
| P0DJD0 | RGPD1 | RGPD1_HUMAN | 1748 | 0.2923 | 0.3471 | 0.3129 | 0.3443 | 0.5097 | 0.5220 | | FALSE | | FALSE | | FALSE |
| P0DJD1 | RGPD2 | RGPD2_HUMAN | 1756 | 0.2995 | 0.3496 | 0.3195 | 0.3464 | 0.5188 | 0.5256 | | FALSE | | FALSE | | FALSE |
| A6NKT7 | RGPD3 | RGPD3_HUMAN | 1758 | 0.2782 | 0.3399 | 0.2958 | 0.3300 | 0.5057 | 0.5180 | | FALSE | | FALSE | | FALSE |
| Q7Z3J3 | RGPD4 | RGPD4_HUMAN | 1758 | 0.3015 | 0.3561 | 0.3134 | 0.3422 | 0.5171 | 0.5320 | | FALSE | | FALSE | | FALSE |

| | | | | | | | | | | | | | | | |
|---|---|---|---|---|---|---|---|---|---|---|---|---|---|---|---|
| Q99666 | RGPD5 | RGPD5_HUMAN | 1765 | 0.2737 | 0.3414 | 0.3286 | 0.3495 | 0.5071 | 0.5126 | | FALSE | | FALSE | | FALSE |
| O14715 | RGPD8 | RGPD8_HUMAN | 1765 | 0.2618 | 0.3383 | 0.3297 | 0.3516 | 0.5065 | 0.5095 | | FALSE | | FALSE | | FALSE |
| Q96DB5 | RMD1 | RMD1_HUMAN | 314 | 0.1401 | 0.2495 | 0.1178 | 0.1713 | 0.3885 | 0.4148 | | FALSE | | FALSE | | FALSE |
| Q9H6T3 | RPAP3 | RPAP3_HUMAN | 665 | 0.3368 | 0.3934 | 0.3744 | 0.4020 | 0.5970 | 0.5833 | 4CGV;4CGW; | TRUE | | FALSE | | FALSE |
| Q8TE82 | S3TC1 | S3TC1_HUMAN | 1336 | 0.1976 | 0.2745 | 0.3331 | 0.3490 | 0.3323 | 0.4090 | | FALSE | | FALSE | | FALSE |
| Q8TF17 | S3TC2 | S3TC2_HUMAN | 1288 | 0.0939 | 0.2178 | 0.1972 | 0.2836 | 0.2065 | 0.3541 | | FALSE | DISEASE: Char | TRUE | | FALSE |
| Q9Y6X3 | SCC4 | SCC4_HUMAN | 613 | 0.0522 | 0.1772 | 0.0734 | 0.1500 | 0.1321 | 0.3020 | | FALSE | | FALSE | | FALSE |
| Q9Y2Z0 | SGT1 | SGT1_HUMAN | 365 | 0.1014 | 0.2610 | 0.2000 | 0.2641 | 0.3918 | 0.4490 | 1RL1; | TRUE | | FALSE | | FALSE |
| O43765 | SGTA | SGTA_HUMAN | 313 | 0.4760 | 0.4941 | 0.5080 | 0.4757 | 0.5655 | 0.5768 | 2VYI;4CPG;4C | TRUE | | FALSE | MUTAGEN 38 3 | TRUE |
| Q96EQ0 | SGTB | SGTB_HUMAN | 304 | 0.3125 | 0.3909 | 0.3289 | 0.3625 | 0.5526 | 0.5161 | | FALSE | | FALSE | | FALSE |
| Q92540 | SMG7 | SMG7_HUMAN | 1137 | 0.5391 | 0.5241 | 0.4644 | 0.4577 | 0.6931 | 0.6472 | 1YA0; | TRUE | | FALSE | MUTAGEN 66 6 | TRUE |
| Q07617 | SPAG1 | SPAG1_HUMAN | 926 | 0.2829 | 0.3661 | 0.4428 | 0.4217 | 0.5248 | 0.5525 | 6I57; | TRUE | DISEASE: Ciliar | TRUE | | FALSE |
| O76094 | SRP72 | SRP72_HUMAN | 671 | 0.2683 | 0.3624 | 0.3562 | 0.3769 | 0.4680 | 0.5239 | 5M72;5M73; | TRUE | DISEASE: Bone | TRUE | | FALSE |
| Q8IZP2 | ST134 | ST134_HUMAN | 240 | 0.4542 | 0.4887 | 0.4792 | 0.5381 | 0.5792 | 0.6023 | | FALSE | | FALSE | | FALSE |
| P31948 | STIP1 | STIP1_HUMAN | 543 | 0.3499 | 0.3807 | 0.2928 | 0.3340 | 0.5580 | 0.5709 | 1ELR;1ELW;2I | TRUE | | FALSE | | FALSE |
| Q9C0D5 | TANC1 | TANC1_HUMAN | 1861 | 0.3987 | 0.4169 | 0.4310 | 0.4493 | 0.5336 | 0.5498 | | FALSE | | FALSE | | FALSE |
| Q9HCD6 | TANC2 | TANC2_HUMAN | 1990 | 0.3879 | 0.4259 | 0.4724 | 0.4787 | 0.5643 | 0.5562 | | FALSE | DISEASE: Note | TRUE | | FALSE |
| Q9Y5Q9 | TF3C3 | TF3C3_HUMAN | 886 | 0.2246 | 0.3079 | 0.3239 | 0.3327 | 0.3002 | 0.4109 | | FALSE | | FALSE | | FALSE |
| Q8IUR5 | TMTC1 | TMTC1_HUMAN | 882 | 0.1168 | 0.2038 | 0.1338 | 0.1826 | 0.2029 | 0.3279 | | FALSE | | FALSE | | FALSE |
| Q8N394 | TMTC2 | TMTC2_HUMAN | 836 | 0.0538 | 0.1743 | 0.0670 | 0.1543 | 0.1555 | 0.2894 | | FALSE | | FALSE | | FALSE |
| Q6ZXV5 | TMTC3 | TMTC3_HUMAN | 915 | 0.0949 | 0.2022 | 0.1355 | 0.1778 | 0.2066 | 0.3221 | | FALSE | DISEASE: Lisse | TRUE | | FALSE |
| Q5T4D3 | TMTC4 | TMTC4_HUMAN | 741 | 0.0404 | 0.1652 | 0.0918 | 0.1259 | 0.1242 | 0.2473 | | FALSE | | FALSE | | FALSE |
| Q15785 | TOM34 | TOM34_HUMAN | 309 | 0.2572 | 0.3203 | 0.3204 | 0.3721 | 0.4822 | 0.5080 | | FALSE | | FALSE | | FALSE |
| O94826 | TOM70 | TOM70_HUMAN | 608 | 0.2525 | 0.3228 | 0.2697 | 0.3164 | 0.3586 | 0.4741 | | FALSE | | FALSE | | FALSE |
| Q96HA7 | TONSL | TONSL_HUMAN | 1378 | 0.3399 | 0.4043 | 0.4565 | 0.4715 | 0.4579 | 0.5550 | 5JA4; | TRUE | | FALSE | | FALSE |
| Q8WVT3 | TPC12 | TPC12_HUMAN | 735 | 0.4247 | 0.4500 | 0.3878 | 0.4322 | 0.4993 | 0.5706 | | FALSE | DISEASE: Ence | TRUE | | FALSE |
| O15050 | TRNK1 | TRNK1_HUMAN | 2925 | 0.1255 | 0.2264 | 0.2530 | 0.2897 | 0.2769 | 0.3765 | | FALSE | | FALSE | | FALSE |
| Q8NDW8 | TT21A | TT21A_HUMAN | 1320 | 0.0258 | 0.1691 | 0.2106 | 0.2503 | 0.1386 | 0.3067 | | FALSE | | FALSE | | FALSE |
| Q7Z4L5 | TT21B | TT21B_HUMAN | 1316 | 0.0403 | 0.1924 | 0.2363 | 0.2849 | 0.1421 | 0.3227 | | FALSE | DISEASE: Note | TRUE | | FALSE |
| Q86WT1 | TT30A | TT30A_HUMAN | 665 | 0.0767 | 0.2127 | 0.2496 | 0.2632 | 0.2256 | 0.3306 | | FALSE | | FALSE | | FALSE |
| Q8N4P2 | TT30B | TT30B_HUMAN | 665 | 0.0707 | 0.2094 | 0.2647 | 0.2730 | 0.2090 | 0.3205 | | FALSE | | FALSE | | FALSE |
| Q5SRH9 | TT39A | TT39A_HUMAN | 613 | 0.0669 | 0.2280 | 0.1338 | 0.2150 | 0.3002 | 0.3770 | | FALSE | | FALSE | | FALSE |
| Q5VTQ0 | TT39B | TT39B_HUMAN | 682 | 0.1540 | 0.2489 | 0.2199 | 0.2541 | 0.2537 | 0.3709 | | FALSE | | FALSE | | FALSE |
| Q8N584 | TT39C | TT39C_HUMAN | 583 | 0.1149 | 0.2304 | 0.1612 | 0.2186 | 0.2642 | 0.3409 | | FALSE | | FALSE | | FALSE |
| Q99614 | TTC1 | TTC1_HUMAN | 292 | 0.6884 | 0.6282 | 0.6062 | 0.5543 | 0.9041 | 0.7753 | | FALSE | | FALSE | | FALSE |
| Q9H892 | TTC12 | TTC12_HUMAN | 705 | 0.1645 | 0.2433 | 0.2142 | 0.2781 | 0.2284 | 0.3814 | | FALSE | | FALSE | | FALSE |
| Q8NBP0 | TTC13 | TTC13_HUMAN | 860 | 0.0814 | 0.2121 | 0.2105 | 0.2519 | 0.2314 | 0.3699 | | FALSE | | FALSE | | FALSE |
| Q96N46 | TTC14 | TTC14_HUMAN | 770 | 0.5273 | 0.5085 | 0.4299 | 0.4539 | 0.7247 | 0.6722 | | FALSE | | FALSE | | FALSE |
| Q8NEE8 | TTC16 | TTC16_HUMAN | 873 | 0.3734 | 0.4279 | 0.4135 | 0.4256 | 0.5258 | 0.5618 | | FALSE | | FALSE | | FALSE |
| Q96AE7 | TTC17 | TTC17_HUMAN | 1141 | 0.1744 | 0.2565 | 0.2743 | 0.3019 | 0.3199 | 0.3944 | | FALSE | | FALSE | | FALSE |
| Q6DKK2 | TTC19 | TTC19_HUMAN | 380 | 0.3526 | 0.3697 | 0.4158 | 0.4344 | 0.4868 | 0.5086 | | FALSE | DISEASE: Mito | TRUE | | FALSE |
| Q5TAA0 | TTC22 | TTC22_HUMAN | 569 | 0.0685 | 0.2311 | 0.2882 | 0.3409 | 0.2373 | 0.3735 | | FALSE | | FALSE | | FALSE |
| Q5W5X9 | TTC23 | TTC23_HUMAN | 447 | 0.1320 | 0.2896 | 0.3512 | 0.3700 | 0.4206 | 0.4492 | | FALSE | | FALSE | | FALSE |
| A2A3L6 | TTC24 | TTC24_HUMAN | 582 | 0.3041 | 0.3997 | 0.3746 | 0.3942 | 0.5979 | 0.5770 | | FALSE | | FALSE | | FALSE |
| Q96NG3 | TTC25 | TTC25_HUMAN | 672 | 0.3095 | 0.3891 | 0.4881 | 0.4480 | 0.5030 | 0.5640 | | FALSE | DISEASE: Ciliar | TRUE | | FALSE |
| Q6P3X3 | TTC27 | TTC27_HUMAN | 843 | 0.0332 | 0.1765 | 0.1578 | 0.2206 | 0.1459 | 0.2993 | | FALSE | | FALSE | | FALSE |
| Q96AY4 | TTC28 | TTC28_HUMAN | 2481 | 0.2851 | 0.3558 | 0.3708 | 0.3967 | 0.4184 | 0.5024 | | FALSE | | FALSE | | FALSE |

| | | | | | | | | | | | | | | | |
|---|---|---|---|---|---|---|---|---|---|---|---|---|---|---|---|
| Q8NA56 | TTC29 | TTC29_HUMAN | 475 | 0.1095 | 0.2326 | 0.2105 | 0.2545 | 0.2611 | 0.3995 | | FALSE | | FALSE | | FALSE |
| P53804 | TTC3 | TTC3_HUMAN | 2025 | 0.2696 | 0.3289 | 0.2973 | 0.3308 | 0.4425 | 0.4995 | | FALSE | | FALSE | MUTAGEN 378 3 | TRUE |
| Q49AM3 | TTC31 | TTC31_HUMAN | 519 | 0.6166 | 0.5416 | 0.5125 | 0.5281 | 0.7360 | 0.7026 | | FALSE | | FALSE | | FALSE |
| Q5I0X7 | TTC32 | TTC32_HUMAN | 151 | 0.2649 | 0.3376 | 0.3245 | 0.3297 | 0.3377 | 0.4142 | | FALSE | | FALSE | | FALSE |
| Q6PID6 | TTC33 | TTC33_HUMAN | 262 | 0.1641 | 0.3002 | 0.3435 | 0.3943 | 0.5305 | 0.4805 | | FALSE | | FALSE | | FALSE |
| A8MYJ7 | TTC34 | TTC34_HUMAN | 566 | 0.1219 | 0.2737 | 0.3569 | 0.3638 | 0.4488 | 0.4601 | | FALSE | | FALSE | | FALSE |
| A6NLP5 | TTC36 | TTC36_HUMAN | 189 | 0.6138 | 0.5736 | 0.5556 | 0.5417 | 0.8148 | 0.6640 | | FALSE | | FALSE | | FALSE |
| Q6PGP7 | TTC37 | TTC37_HUMAN | 1564 | 0.0384 | 0.1765 | 0.1522 | 0.2031 | 0.1611 | 0.3230 | | FALSE | DISEASE: Trich | TRUE | | FALSE |
| Q5R3I4 | TTC38 | TTC38_HUMAN | 469 | 0.0618 | 0.1877 | 0.1578 | 0.1964 | 0.1365 | 0.3197 | | FALSE | | FALSE | | FALSE |
| O95801 | TTC4 | TTC4_HUMAN | 387 | 0.2610 | 0.3340 | 0.3385 | 0.3590 | 0.4677 | 0.4841 | 6HFO; | TRUE | | FALSE | MUTAGEN 42 42 | TRUE |
| Q6P2S7 | TTC41 | TTC41_HUMAN | 1318 | 0.0797 | 0.1966 | 0.1305 | 0.1870 | 0.2375 | 0.3561 | | FALSE | | FALSE | | FALSE |
| Q8N0Z6 | TTC5 | TTC5_HUMAN | 440 | 0.1591 | 0.2995 | 0.2705 | 0.3309 | 0.5227 | 0.4884 | 2XVS; | TRUE | | FALSE | | FALSE |
| Q86TZ1 | TTC6 | TTC6_HUMAN | 520 | 0.0500 | 0.1712 | 0.0308 | 0.1105 | 0.1308 | 0.2678 | | FALSE | | FALSE | | FALSE |
| Q9ULT0 | TTC7A | TTC7A_HUMAN | 858 | 0.1154 | 0.2560 | 0.2552 | 0.3075 | 0.2984 | 0.3989 | | FALSE | DISEASE: Gastr | TRUE | | FALSE |
| Q86TV6 | TTC7B | TTC7B_HUMAN | 843 | 0.0973 | 0.2327 | 0.2847 | 0.3344 | 0.2313 | 0.3603 | 5DSE;6BQ1; | TRUE | | FALSE | | FALSE |
| Q8TAM2 | TTC8 | TTC8_HUMAN | 541 | 0.1904 | 0.2892 | 0.2514 | 0.2744 | 0.2643 | 0.3819 | | FALSE | DISEASE: Retin | TRUE | | FALSE |
| Q92623 | TTC9A | TTC9A_HUMAN | 222 | 0.4717 | 0.4550 | 0.5090 | 0.5145 | 0.6216 | 0.5948 | | FALSE | | FALSE | | FALSE |
| Q8N6N2 | TTC9B | TTC9B_HUMAN | 239 | 0.5439 | 0.5516 | 0.5649 | 0.5686 | 0.7029 | 0.6506 | | FALSE | | FALSE | | FALSE |
| Q8N5M4 | TTC9C | TTC9C_HUMAN | 171 | 0.4269 | 0.4556 | 0.5263 | 0.5011 | 0.5848 | 0.5418 | | FALSE | | FALSE | | FALSE |
| Q9H3U1 | UN45A | UN45A_HUMAN | 944 | 0.0477 | 0.2224 | 0.3273 | 0.3626 | 0.1642 | 0.3576 | 2DBA; | TRUE | | FALSE | MUTAGEN 33 33 | TRUE |
| Q8IWX7 | UN45B | UN45B_HUMAN | 931 | 0.0473 | 0.1898 | 0.1794 | 0.2739 | 0.1772 | 0.3365 | | FALSE | DISEASE: Catar | TRUE | | FALSE |
| O14607 | UTY | UTY_HUMAN | 1347 | 0.3088 | 0.3425 | 0.2725 | 0.2968 | 0.4447 | 0.4761 | 3ZLI;3ZPO;4U | TRUE | | FALSE | MUTAGEN 1093 | TRUE |
| Q96RL7 | VP13A | VP13A_HUMAN | 3174 | 0.0548 | 0.1934 | 0.1994 | 0.2436 | 0.2023 | 0.3387 | | FALSE | DISEASE: Chor | TRUE | | FALSE |
| Q8NEZ3 | WDR19 | WDR19_HUMAN | 1342 | 0.0201 | 0.1558 | 0.1244 | 0.1769 | 0.1185 | 0.2769 | | FALSE | DISEASE: Crani | TRUE | | FALSE |
| Q8N5D0 | WDTC1 | WDTC1_HUMAN | 677 | 0.1610 | 0.2464 | 0.2171 | 0.2617 | 0.2866 | 0.3865 | 3I7N; | TRUE | | FALSE | | FALSE |
| Q8IWR0 | Z3H7A | Z3H7A_HUMAN | 971 | 0.2142 | 0.3024 | 0.3007 | 0.3316 | 0.4274 | 0.4524 | 2D9M; | TRUE | | FALSE | | FALSE |
| Q9UGR2 | Z3H7B | Z3H7B_HUMAN | 977 | 0.2692 | 0.3446 | 0.3889 | 0.3819 | 0.5159 | 0.5139 | | FALSE | | FALSE | MUTAGEN 241 2 | TRUE |
| O60293 | ZC3H1 | ZC3H1_HUMAN | 1989 | 0.4681 | 0.4735 | 0.4374 | 0.4430 | 0.5892 | 0.6166 | | FALSE | | FALSE | | FALSE |
| Q9H0C1 | ZMY12 | ZMY12_HUMAN | 365 | 0.0932 | 0.2103 | 0.2384 | 0.2279 | 0.1699 | 0.2865 | | FALSE | | FALSE | | FALSE |
